# Routing the Exciton Emissions of WS_2_ Monolayer with the High-Order Plasmon Modes of Ag Nanorods

**DOI:** 10.1021/acs.nanolett.3c00054

**Published:** 2023-05-09

**Authors:** Shasha Li, Ruoqi Ai, Ka Kit Chui, Yini Fang, Yunhe Lai, Xiaolu Zhuo, Lei Shao, Jianfang Wang, Hai-Qing Lin

**Affiliations:** † Beijing Computational Science Research Center, Beijing 100193, People’s Republic of China; ‡ Department of Physics, The Chinese University of Hong Kong, Shatin, Hong Kong SAR 999077, People’s Republic of China; § State Key Laboratory of Optoelectronic Materials and Technologies, Guangdong Province Key Laboratory of Display Material and Technology, School of Electronics and Information Technology, Sun Yat-sen University, Guangzhou 510275, People’s Republic of China; ∥ School of Science and Engineering, The Chinese University of Hong Kong (Shenzhen), Shenzhen 518172, People’s Republic of China

**Keywords:** high-order plasmon modes, light routing, plasmon−exciton interaction, silver nanorods, tungsten disulfide monolayer

## Abstract

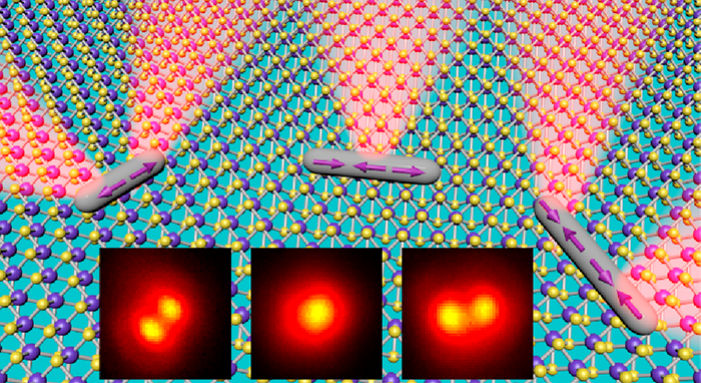

Locally routing the exciton emissions in two-dimensional (2D) transition-metal dichalcogenides along different directions at the nanophotonic interface is of great interest in exploiting the promising 2D excitonic systems for functional nano-optical components. However, such control has remained elusive. Herein we report on a facile plasmonic approach for electrically controlled spatial modulation of the exciton emissions in a WS_2_ monolayer. The emission routing is enabled by the resonance coupling between the WS_2_ excitons and the multipole plasmon modes in individual silver nanorods placed on a WS_2_ monolayer. Different from prior demonstrations, the routing effect can be modulated by the doping level of the WS_2_ monolayer, enabling electrical control. Our work takes advantage of the high-quality plasmon modes supported by simple rod-shaped metal nanocrystals for the angularly resolved manipulation of 2D exciton emissions. Active control is achieved, which offers great opportunities for the development of nanoscale light sources and nanophotonic devices.

The control of light propagation direction is essential in modern optics that uses light or photons as the medium for carrying and processing information. Subwavelength plasmonic nanoantennas, which can change the propagation path of light energy or route electromagnetic radiation into certain directions in subdiffraction-limit volumes,^1,2^ are important candidates for achieving the ultimate spatial control of light. They support a range of advanced light manipulation capabilities, including unidirectional light emission, polarization conversion, and fluorescence enhancement.^3−5^ Most research works thus far on optical nanoantennas have concentrated on spatially dependent static light control. Active modulation of emission routing with optical nanoantennas is of great interest in constructing dynamic electro-optical components^6,7^ but has remained elusive. The integration of optical nanoantennas with excitonic materials is promising for achieving this goal. On the one hand, the *in situ* integration of an electrically driven light source is allowed with the use of excitonic materials. On the other hand, dynamically modulating the optical response of the coupling system is possible with external stimuli.^8^ This can not only introduce new functionalities into excitonic systems but also establish viable approaches to the design of key components, such as optical modulators, optical switches, and nanoscale light sources, for on-chip nanophotonic circuits.^1,9−11^

Two-dimensional (2D) transition-metal dichalcogenides (TMDCs) with unique optical features, such as large binding energies,^12^ entangled valley-spin degrees of freedom,^13,14^ and rich excitonic complexes,^15^ have become some of the most promising excitonic materials for a range of applications in nanophotonics and optoelectronics.^16−18^ The integration of 2D TMDCs with nanophotonic structures is extremely important for overcoming the intrinsically weak light–matter interaction of the atomic layer structure.^19,20^ In comparison with the intensity modulation,^21,22^ which has been extensively studied, the spatial modulation of the exciton emissions in 2D TMDCs has so far received much less attention. A few works in this aspect have employed Mie resonance modes in high-index dielectric structures for directional forward emissions of 2D excitons^23,24^ and optical spin–orbit coupling in metamaterials,^25,26^ as well as surface plasmon polaritons^27−29^ for spatially separated transport of the valley emissions in 2D TMDCs. Nanoantenna structures that have a small footprint and can achieve effective emission routing in 2D TMDCs are highly desired but have remained lacking. Moreover, the realization of electrical control of the emission routing in 2D TMDCs is also highly attractive for developing functional optoelectronic devices.

A promising opportunity is provided by coupling 2D excitons with multipole plasmon resonance modes. In contrast to well-known dipole plasmon modes with well-defined spatial emissions, multipole plasmon resonance modes that give angularly resolved radiation are usually considered as “dark modes”, because they are hard to excite by propagating plane waves owing to their high symmetry.^30−32^ It has been proposed that precisely positioning a point optical source, such as an electric dipole source, at a plasmonic “hot spot” is advantageous for breaking the symmetry and enabling access to subradiant modes by far-field illumination.^5^ Such a strategy has been explored for simultaneously enhancing the emission intensity, directionality, and polarization of quantum dots and fluorescent molecules.^5,33−35^ However, new challenges emerge when 2D excitons are involved. First, it is challenging to precisely position 2D excitons at the specific position on a plasmonic nanoantenna to obtain the strongest modulation. The large background signal from the portion of the 2D monolayer that does not interact with the nanoantenna also hinders the observation of the emission directionality. Second, alignment of the emission dipoles with plasmonic multipoles is difficult because the excitons in 2D TMDCs suffer from an ultrafast decoherence process and orient randomly in the 2D plane.^36,37^ Whether high-order plasmon resonance modes can effectively interact with 2D excitons has therefore remained an open question.

In this work we demonstrate the effective emission routing in a WS_2_ monolayer with Ag nanorods (NRs) by taking advantage of the angularly resolved radiation of the multipole plasmon resonance modes with even symmetries. Different from previous reports, the emission routing effect in our structures can be modulated by electron doping in the WS_2_ monolayer. The underlying mechanism has been systematically studied through electrodynamic simulations and polarization-dependent spectroscopy measurements, which confirm the important roles of the resonance coupling in the plasmon–exciton system and the photoluminescence (PL) enhancement difference between the neutral and charged excitons. A proof-of-concept device is designed for electrical control of the routing of the exciton emissions. Our results provide a good platform to achieve the electrically controllable modulation of the emission directionality of 2D TMDC excitons, offering great possibilities for the future design of on-chip integrated nanophotonic devices such as high-performance nanorouters, optical switches, and directional nanoscale light sources.

We prepared (Ag NR)-on-WS_2_ structures by depositing Ag NRs onto a piece of WS_2_ monolayer grown on a quartz substrate (Figure 1a–c; see Methods in the Supporting Information). The plasmonic Ag NRs synthesized through a wet-chemistry method exhibit excellent size uniformity and high crystallinity (Figure 1a and Figure S1).^31^ The high-aspect-ratio Ag NRs can support high-quality multipole plasmon modes with odd and even symmetries, whose spectral positions are dependent on the length of the Ag NR (Figure S2).^3^ The WS_2_ monolayer was directly grown on quartz substrates, showing a pronounced PL peak at ∼630 nm (Figure S3a,b). The dark-field scattering characteristics of the multipole plasmon resonance modes in the Ag NRs are well preserved by use of low-refractive-index substrates (Figures S2 and S3c–e), which is beneficial for the following spectroscopic studies. The multipole plasmon modes (*N* = 2, 3, 4) of the Ag NRs were synthetically adjusted to resonantly couple with the exciton transitions in the WS_2_ monolayer (Figure 1d). A sharp dip located at the transition energy of the A excitons was observed from the dark-field scattering spectra. Sharp dips have also been observed in works on the resonance coupling between the dipole plasmon mode and 2D excitons.^38,39^ The mode analysis based on a coupled oscillator model shows that the obtained splitting energies (ℏΩ) are slightly larger than that required for strong coupling, i.e., ℏΩ > (ℏγ_pl_ + ℏγ_ex_)/2, in all three cases (Figure 1e, Figures S4 and S5). Herein (ℏγ_ex_+ ℏγ_pl_)/2 refers to the overall loss of the system, with ℏγ_ex_ and ℏγ_pl_ being the line widths of the exciton emissions and the involved high-order plasmon resonance mode, respectively. The narrow line widths of the high-order plasmon modes lead to low-loss plasmon–exciton coupling. Our results demonstrate that the high-order plasmon modes in the Ag NRs can strongly couple to the 2D TMDC excitons, offering great opportunities for exploring new functionality enabled by the multipole plasmon characteristics. Given the far-field angular scattering behaviors of the Ag NRs (Figures S2 and S3e), the plasmon–exciton coupling in these systems enables the plasmon-modulated directional emissions from the WS_2_ monolayer (Figure 1f,g), which will be discussed below.

**Figure 1:**
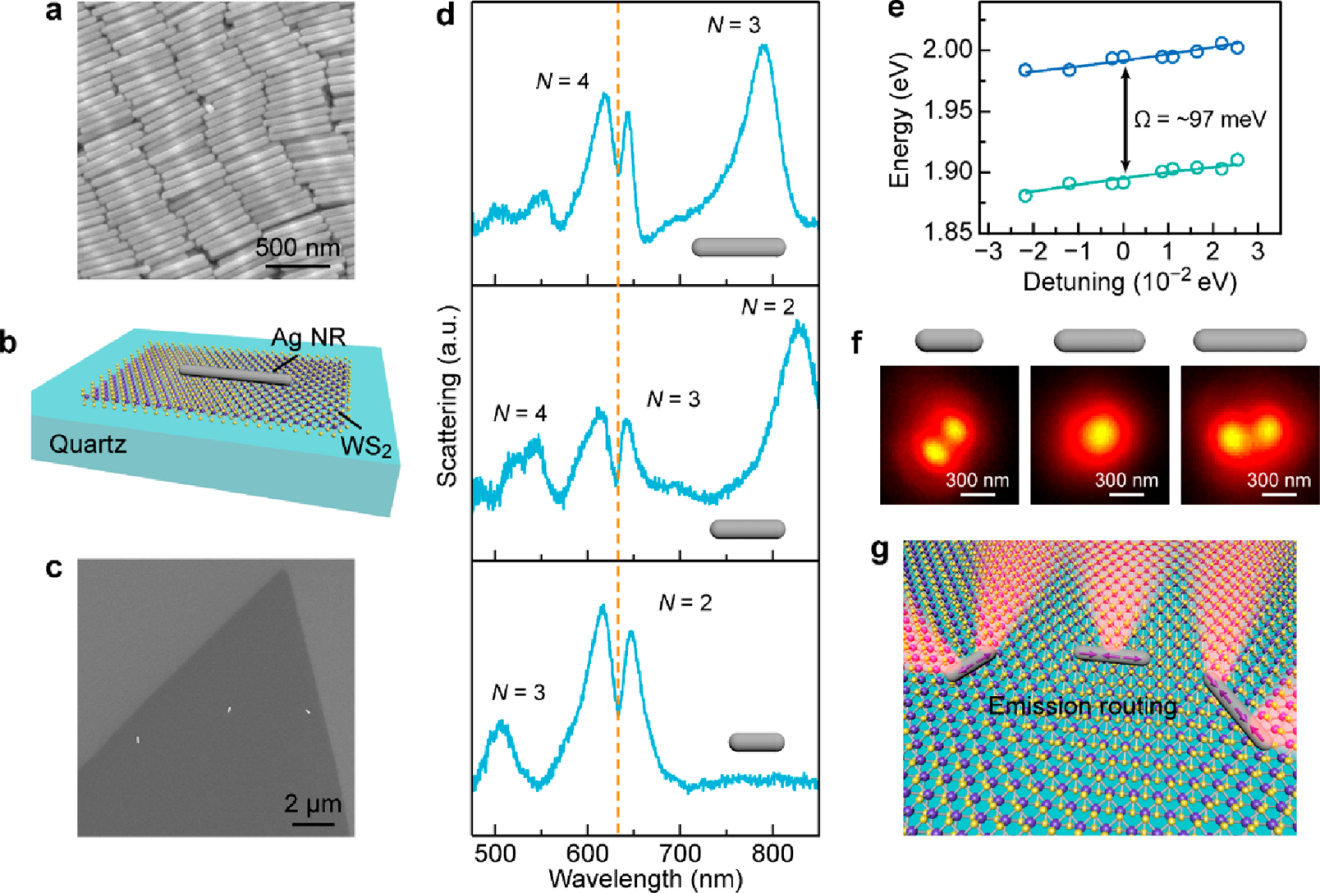
WS_2_ monolayer resonantly coupled to the multipole plasmon modes in the Ag NRs. (a) Scanning electron microscopy (SEM) image of a representative Ag NR sample. The diameter and length of the displayed Ag NRs are 58 ± 4 and 490 ± 21 nm, respectively. (b) Schematic showing a (Ag NR)-on-WS_2_ heterostructure supported on a quartz substrate. (c) SEM image captured after the Ag NRs were deposited on the WS_2_ monolayer grown on a quartz substrate. The Ag NRs are well dispersed with a particle-to-particle distance larger than 5 μm, which is beneficial for the single-particle measurements. (d) Dark-field scattering spectra of the (Ag NR)-on-WS_2_ heterostructures, where the lengths of the Ag NRs were adjusted for resonantly coupling the 2D excitons with the different multipole plasmon modes in the Ag NRs. The used Ag NR samples have a similar average diameter of 58 ± 4 nm and three different average lengths, which are 310 ± 15, 490 ± 21, and 601 ± 38 nm, respectively. (e) Energy dependence of the scattering peaks on the detuning. The coupling between the quadrupole plasmon mode (*N* = 2) and the exciton transition is illustrated as a representative result. The mode-splitting energy at zero detuning (∼97 meV) is slightly larger than the overall loss of the system, suggesting strong plasmon–exciton coupling. Herein ℏγ_ex_ = 52 meV and ℏγ_pl_ = 135 meV are employed for the estimation. (f) PL images of the (Ag NR)-on-WS_2_ heterostructures with the multipole plasmon modes resonantly coupled to the exciton transition. The PL images were captured and redrawn with the same scale bar in this work. (g) Schematic showing the spatial modulation of the exciton emissions in the WS_2_ monolayer.

The WS_2_ monolayer was inevitably doped during the sample preparation process. For example, the residual cetyltrimethylammonium chloride (CTAC) and water in the Ag NR solution are two well-known n-type dopants to a WS_2_ monolayer.^40,41^ The highly doped WS_2_ monolayer exhibits a substantially decreased PL intensity and an increased trion-to-exciton ratio (Figure 2a,b). The (Ag NR)-on-WS_2_ heterostructures constructed from the slightly and highly doped WS_2_ monolayers on quartz substrates show elongated emission patterns with distinct features (Figure 2c–f). The emission patterns of the slightly doped samples appear as a solid bright spot, while those of the highly doped samples show two separate bright spots. The above phenomena were observed in both types of structures, where the exciton emissions are resonant to the *N* = 2 and *N* = 4 plasmon modes, respectively (Figure S6). Similar PL emission patterns were also obtained from the (Ag NR)-on-WS_2_ heterostructures prepared on Si/SiO_2_ substrates (Figures S7 and S8). The exciton emissions with a narrow line width in our structures were not affected by the interference from the thermal oxide layer, which has been reported to alter the dark-field scattering spectra of Ag NRs in our previous work.^3^ The heterostructures supported on the Si/SiO_2_ substrates will be employed for further investigation for two reasons. First, the WS_2_ monolayer grown on the Si/SiO_2_ substrates possesses a larger grain area and is more tightly attached to the substrate. Second, the Si/SiO_2_ substrate is advantageous for electron microscopy characterization.

**Figure 2:**
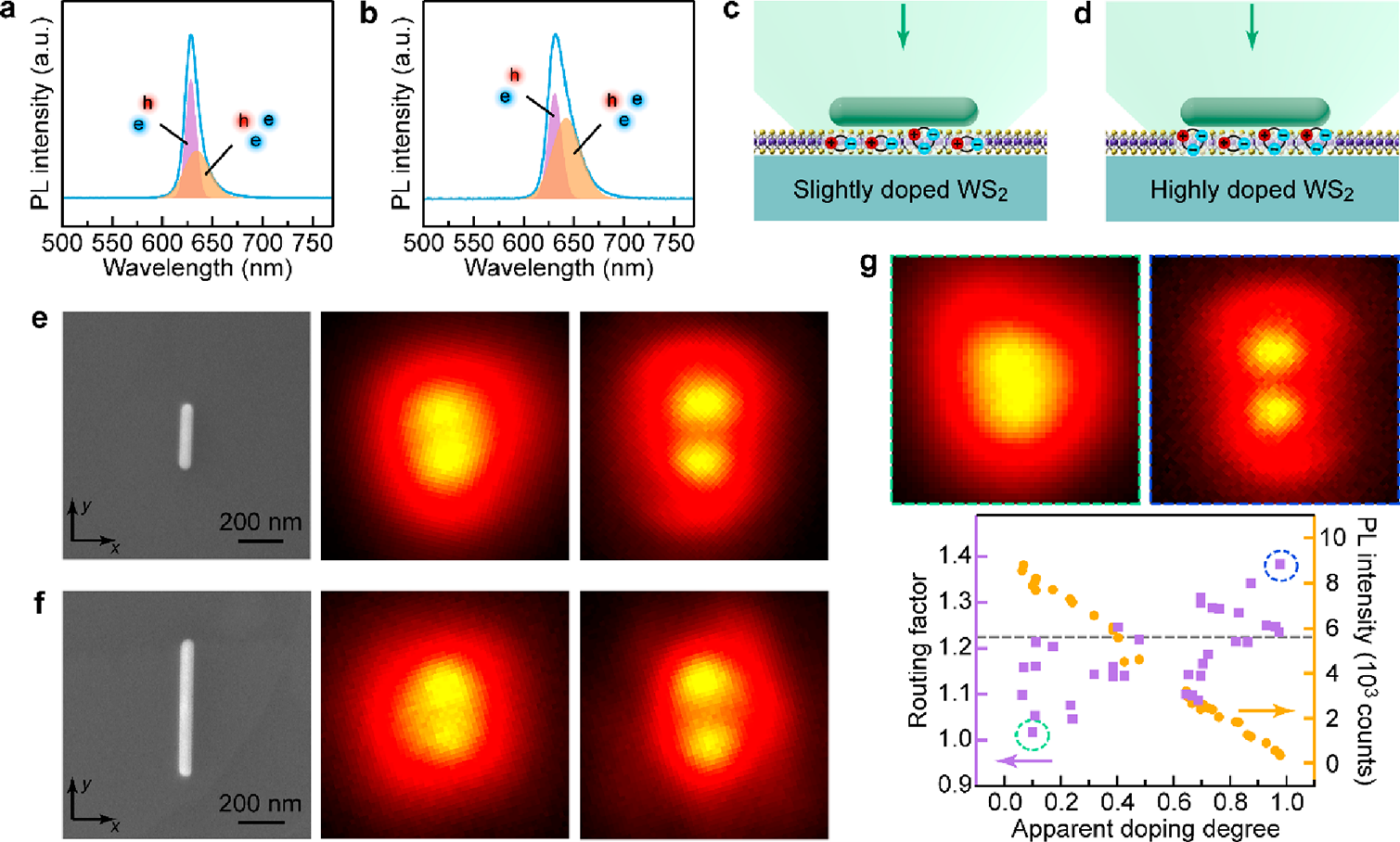
Directional exciton emissions modulated by the even plasmon modes. (a, b) Representative PL spectra obtained from a (a) slightly and a (b) highly doped WS_2_ monolayer under 514 nm laser excitation. The PL peaks of the A excitons and trions were extracted by Gaussian fitting. (c, d) Schematics showing the (Ag NR)-on-WS_2_ heterostructures constructed from a slightly (c) and highly (d) doped WS_2_ monolayer. (e, f) SEM (left) and PL images of the heterostructures in the slightly (middle) and highly (right) doped cases. The used Ag NR samples have an average length of 310 nm in (e) and 601 nm in (f), respectively. The exciton emissions are correspondingly on-resonance with the *N* = 2 (e) and *N* = 4 (f) plasmon modes, respectively. A clear PL routing effect can only be seen on the highly doped WS_2_ monolayer. (g) Dependence of the routing factor and the integrated PL intensity on the apparent doping degree. The data were collected from the (Ag NR)-on-WS_2_ heterostructures prepared on the Si/SiO_2_ (300 nm thickness) substrates. The used Ag NR sample has an average length of 310 nm. The typical PL images corresponding to the two extreme cases are shown on the top. The gray dashed line indicates the scattering routing factor value of the *N* = 2 plasmon resonance. The SEM and PL images in (e–g) are shown in the same coordinate reference. The intensity data of the PL images are normalized for a better illustration of the routing patterns.

The double bright spots in the emission patterns were experimentally verified to be distributed along the length axis of the Ag NR through correlating the PL image with the orientation of each Ag NR observed under SEM (Figure S9). The above results strongly indicate that the exciton emissions in the WS_2_ monolayer are rerouted toward the two ends of the NR by the even plasmon modes. More interestingly, the PL routing effect is significantly enhanced by electron doping of the WS_2_ monolayer. In a quantitative analysis, the emission anisotropy was found to be proportionally modulated by the level of electron doping in the WS_2_ monolayer (Figure 2g). The emission anisotropy is evaluated by the routing factor, which is defined as the peak-to-dip intensity ratio along the longitudinal intensity profile extracted from the emission pattern (Figure S10). Electron doping in the WS_2_ monolayer is characterized by the apparent doping degree, which is expressed as 1 – *I*_a_/*I*_b_, with *I*_a_ and *I*_b_ representing the integrated PL intensities measured from the same piece of a WS_2_ monolayer after and before the sample preparation process due to the involvement of CTAC and/or water, respectively. The scattering routing factors for both *N* = 2 and *N* = 4 modes were also determined (Figure S11). The PL routing factor can be adjusted by electron doping, with the values in the highly doped structures exceeding the scattering routing factor. The difference between the dipole emission directionality and the plane wave scattering directionality has also been observed in previous works.^23,42^ The modified resonance constributions and the interference between the incident field from the dipole sources and the scattered field make the localized source excitation different from the plane wave case. Such a strong PL routing effect occurs with no need of precise positioning of nanoemitters and self-aligned etching of background WS_2_, as demonstrated in previous studies.^5,23,33^ The enhanced emission routing can also be observed in the doped system realized by other n-type dopants (Figure S12). The total photon emissions of the heterostructures decrease in intensity with increasing apparent doping degrees. Chemical doping through molecular adsorption can introduce nonradiative channels into the WS_2_ monolayer and is unfavorable for the formation of radiative trions,^43^ which leads to the reduced photon emissions.

To ascertain the physical origin of the observed emission routing, finite-difference time-domain (FDTD) simulations were performed to analyze the near- and far-field properties of the plasmon–exciton system. A 1 nm thick layer inserted between the nanorod and the substrate was used to model the dielectric environment in the presence of the WS_2_ monolayer. The length of the nanorod in the model was adjusted to align the simulated scattering peaks with the measured ones (Figures S13 and S14). The near-field images clearly demonstrate the existence of the multipole plasmon resonance modes with even symmetries (Figure 3a,b). Such multipole plasmon modes are able to guide the radiation path of nanoemitters placed nearby, which is confirmed by the far-field radiation patterns composed of two broad lobes (Figure 3c,d). Our simulation suggests that although the excitons of the WS_2_ monolayer appear in a 2D plane, the angular far-field radiation is dominated by the *x*-polarized emitter located at the ends of the nanorod (Figures S15 and S16). This is reasonable because of the strong local field enhancement at the ends of the nanorod and the fact that the dipole moment is aligned well with the local electric field of the longitudinal plasmon mode of the nanorod. The radiation directionality is highly sensitive to the resonance coupling and the dipole–antenna distance (Figures S17 and S18), similar to previous studies.^44,45^ We also investigated the intrinsic loss induced by the Ag NR (Table S1). The plasmonic nanostructures introduce a large energy loss, compared to dielectric nanoantennas.^23,42,46^ The addition of an insulator gap layer can help to suppress the metal loss,^47^ which requires further study.

**Figure 3:**
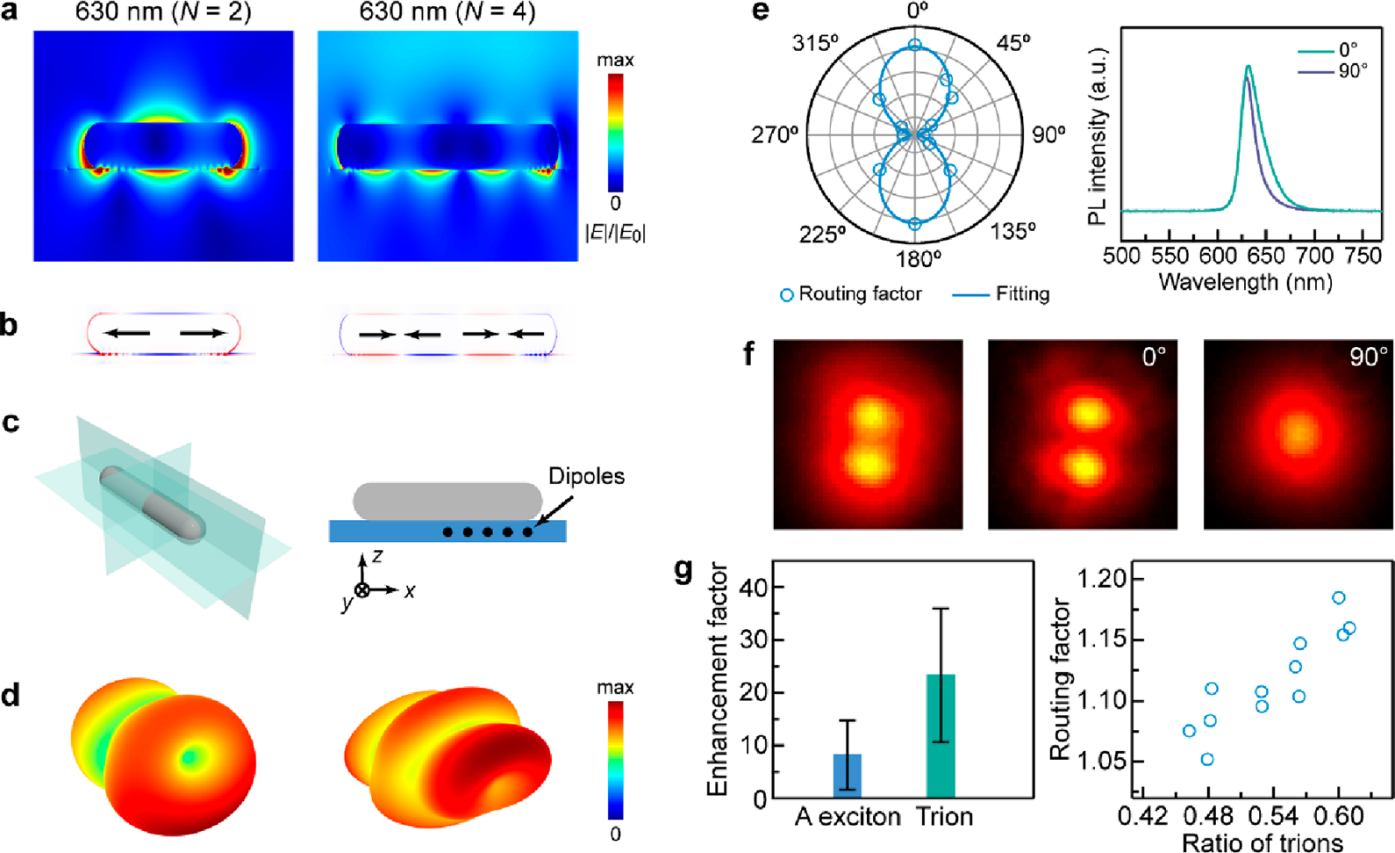
Mechanisms for the enhanced routing effect. (a, b) Simulated electric field distribution (a) and charge distribution (b) contours of the even plasmon resonance modes. The lengths of the nanorods in the model are 305 and 640 nm. (c) Schematics showing the spatial orientation of the nanorod (left) and the emitter positions (right) relative to the nanorod in the far-field radiation simulation. (d) Total far-field radiation arising from the dipole emissions from the five representative positions shown in (c). The dipoles orientated along the three orthogonal axes of the displayed coordinate system were considered. (e) Emission polarization dependence. The left panel shows the polarization polar plot of the routing factor as a function of the analyzer polarization direction. The polar plot was fitted by a sine-squared function. The right panel displays the PL spectra obtained at the polarization angles of 0 and 90°, respectively. (f) PL images of a representative structure captured without (left) and with an analyzer in front of the entrance of the camera aligned at 0° (middle) and 90° (right). (g) Influence of the PL enhancement on the emission routing. The PL enhancement factors of the A excitons and trions are illustrated in the left panel. The routing factor is presented as a function of the trion ratio in the right panel.

The distinct routing effect induced by electron doping suggests that the nature of the nanoemitters also affects the radiation properties, which cannot be reflected by the FDTD simulations. To further explore the mechanism for the enhanced directional emissions, polarization-dependent PL measurements were conducted (Figure 3e, Figures S19 and S20). The PL routing effect was found to be independent of the excitation polarization (Figure S19), indicating that the excited excitons are orientated randomly in the 2D plane due to the ultrafast decoherence process.^36,37^ The emission polarization dependence was investigated by inserting a linear polarizer in front of the entrance of the camera. The recorded PL routing effect was found to be most significant when the analyzer polarization was aligned parallel to the length axis of the Ag NR, while it disappeared when the analyzer polarization was perpendicular to the length axis (Figure 3e and Figure S20). The exciton emissions are clearly endowed with the polarization-dependent property of the longitudinal multipole plasmon resonance modes due to strong plasmon–exciton coupling. We also note that the anisotropic emission pattern with two bright spots can only be seen when the plasmon-modulated PL emissions are stronger than the background signal originating from the bare WS_2_ monolayer (Figure 3f and Figure S21).

The polarization-dependent spectroscopy measurements also provide an approach to study the plasmon-enabled PL enhancement of the two types of 2D excitons: i.e., A excitons and trions. The PL enhancement factor defined as the ratio of the plasmon-modulated signals and the background was employed for analysis (Figure S21). The obtained PL enhancement factor of the trions is much higher than that of the A excitons (Figure 3g, left). In a piece of a highly doped WS_2_ monolayer, a large portion of the A excitons are converted into trions, leading to an enhanced plasmon-modulated routing effect (Figure 3g, right). The enhanced routing effect of the trions can also be seen clearly by separating the contributions of the A excitons and trions (Figure S22). The larger PL enhancement for the trions probably results from the following reason. The trions possess both in-plane and out-of-plane emission dipole moments,^48,49^ while the A excitons only have the in-plane moments. The out-of-plane emission dipole component enables additional coupling with the excited near-field and will also contribute to the PL enhancement (Figures S15 and S16). The larger PL enhancement of the trions by the plasmonic near-field is critical for the observation of the more distinct routing effect of the PL signal. Our results demonstrate for the first time the importance of the trions for emission routing.

The difference in the plasmon-enabled PL enhancement of the A excitons and trions opens up new opportunities for electrical control of the emission routing effect (Figure 4). The proof-of-concept structure was fabricated on the Si/SiO_2_ substrates, with the Si layer as the back gate (Figure 4a and Figure S23). The electron doping of the WS_2_ monolayer is well-known to be enhanced under a positive gate bias and suppressed under a negative bias, respectively. As a result, the PL of the WS_2_ monolayer can be adjusted (Figure S24), indicating the change of the exciton-to-trion ratio. The emission routing effect was therefore enhanced at a positive gate bias, as confirmed by a more distinct ∞-shaped pattern (Figure 4b). The dynamic modulation process was further performed by switching the gate voltage and recorded by a color camera (Figure 4c). The PL emissions of an individual (Ag NR)-on-WS_2_ heterostructure can be reversibly switched between the ∞-shaped pattern and a bright solid spot. This indicates that the (Ag NR)-on-WS_2_ structure can act as an “electrically controlled router” to guide the radiation energy toward the two ends of the nanorod. Although our results were demonstrated under the optical excitation, it is reasonably expected that a similar effect can also be achieved through electroluminescence excitation: for example, in a light-emitting diode structure. It is also believed that the routing strategy can be employed for the spatial modulation of 2D excitons with out-of-plane dipole moments in addition to trions, such as dark excitons, interlayer excitons, and localized excitons. This can benefit diverse nanophotonic applications from quantum information technologies to valley-spin optoelectronics.^50−52^ We note that the trion emissions in our study should be optimized for possible future applications. The trion emissions can be improved through other doping approaches, such as interlayer doping using the type-I band alignment in van der Waals heterostructures.^53^ The enhanced radiative decay of trions will also suppress the nonradiative channel and lead to narrowed exciton line widths.^54^

**Figure 4:**
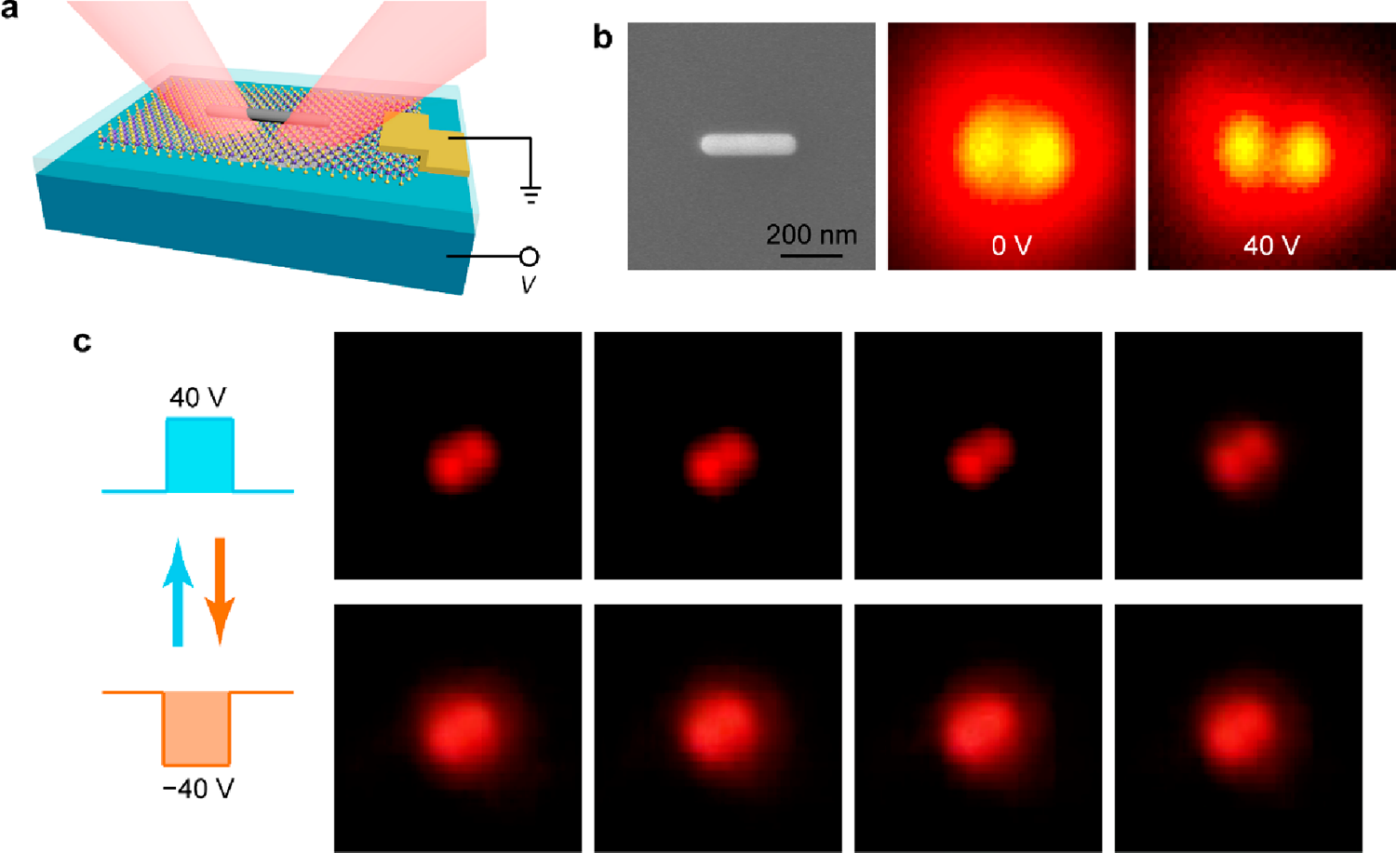
Electrical modulation of the PL routing. (a) Schematic showing the electrically modulated (Ag NR)-on-WS_2_ heterostructure. The Si layer was employed as the back gate, while the WS_2_ monolayer was grounded. (b) SEM image and PL images of a representative structure. The emission routing was enhanced at a positive gate voltage, which causes electron doping in the WS_2_ monolayer. (c) Robust and dynamic control of the emission routing effect by the electrical means. The PL images were captured with a color camera.

In summary, we have demonstrated the efficient spatial modulation of the exciton emissions in a WS_2_ monolayer with Ag nanorods as nanoantennas. Such a modulation of the emission directionality is enabled by the resonance coupling between the 2D excitons and the multipole plasmon resonance modes with even symmetries. The exciton emissions can be directed to the two ends of the nanorod. An enhanced routing effect has been achieved in highly doped WS_2_ monolayer, which is attributed to the large plasmon-enabled PL enhancement of the negatively charged trions formed from the neutral excitons and electrons. A proof-of-concept device is employed to electrically control the emission direction of the 2D excitons. Our work establishes a new hybrid plasmon–exciton system for the spatial modulation of the exciton emissions from 2D TMDCs with active control. Our strategy opens new opportunities for the future design of on-chip integrated nanophotonic devices such as high-performance nanorouters, optical switches, and directional nanolight sources. It also holds great promise for a range of applications requiring tight confinement of optical fields, including sensing, high-performance photodetection, and nano-optical spectroscopy.

## Supporting Information Available

Methods for the sample preparation and characterization, optical measurements, and simulations, Ag NR samples, (Ag NR)-on-WS_2_ heterostructures, resonance coupling in the (Ag NR)-on-WS_2_ heterostructures, calculation of the PL routing factor, scattering routing factor, PL routing results after chemical modification, FDTD simulation results, excitation and emission polarization dependence, electrically biased (Ag NR)-on-WS_2_ heterostructures, and electrically modulated PL emissions of the WS_2_ monolayer (PDF)
