# Supplementary material for: Routing the Exciton Emissions of WS2 Monolayer with the High-Order Plasmon Modes of Ag Nanorods
Source: Nano Lett. 2023 May 9;23(10):4183–90. doi: 10.1021/acs.nanolett.3c00054 (PMC10214448; doi:10.1021/acs.nanolett.3c00054)
Supplement: Supplementary file 1 — nl3c00054_si_001.pdf [file nl3c00054_si_001.pdf]

Supporting Information

Routing the Exciton Emissions of WS_2 Monolayer with the High-Order Plasmon Modes of Ag Nanorods

Shasha Li,[1,2,§] *Ruoqi Ai,*[2,§] *Ka Kit Chui,*[2] *Yini Fang,*[2] *Yunhe Lai,*[2] *Xiaolu Zhuo,*[4] *Lei Shao,*[3,*] *Jianfang Wang,*[2,*] *and Hai-Qing Lin*[1]

[1]Beijing Computational Science Research Center, Beijing 100193, China.

[2]Department of Physics, The Chinese University of Hong Kong, Shatin, Hong Kong SAR 999077, China.

[3]State Key Laboratory of Optoelectronic Materials and Technologies, Guangdong Province Key Laboratory of Display Material and Technology, School of Electronics and Information Technology, Sun Yat-sen University, Guangzhou 510275, China.

[4]School of Science and Engineering, The Chinese University of Hong Kong (Shenzhen), Shenzhen 518172, China.

*Email: shaolei5@mail.sysu.edu.cn; jfwang@phy.cuhk.edu.hk

[§]S.S.L. and R.Q.A. contributed equally to this work.

METHODS

Sample Preparation and Characterization. The Ag NRs were synthesized by a Au-nanobipyramid-directed Ag overgrowth method.[1,2] The diameter of the Ag NRs was controlled by the waist diameter of the Au nanobipyramids. The length of the Ag NRs was adjusted by changing the amount of the $AgNO_3$ precursor in the overgrowth process. The WS_2 monolayer was grown on quartz and Si/SiO_2 substrates at temperatures of 1170–1200 °C by physical vapor deposition.[3] The (Ag NR)-on-WS_2 heterostructures were fabricated by dropping the solution of the washed Ag NRs onto the quartz or Si/SiO_2 substrates with the pre-grown WS_2 monolayer and drying it with N_2. The gated (Ag NR)-on-WS_2 heterostructures were fabricated on Si/SiO_2 substrates. The electrode was made as follows. A piece of Au film evaporated on a Si substrate was first peeled off with a polydimethylsiloxane (PDMS) film and transferred to cover a part of the WS_2 monolayer on a home-built transfer system. The PDMS film was peeled off from the substrate at 90 °C. The Ag NRs were then deposited on the WS_2 monolayer with the Au electrode. The morphological characterization was conducted on a scanning electron microscope (JEOL, JSM-7800F) operated at 10 kV.

Single-Particle Optical Measurements. The PL and dark-field scattering measurements were performed on an upright optical microscope (Olympus, BX51) equipped with a monochromator (Acton, SpectraPro 2360i), a charge-coupled device camera (Princeton Instruments, Pixis 400, cooled to –70 °C), and a color camera (Olympus, DP73). A 514 nm argon ion laser (Spectra-Physics, Stabilite 2017) was introduced into the optical microscope for focusing and used to optically excite the nanostructures. The power of the laser was set at ~1 μW. A 550 nm long-pass filter was employed to block the laser reflected/scattered from the substrate. A 100× dark-field objective (numerical aperture 0.9) was employed for both PL and dark-field scattering measurements. The polarization-resolved PL measurements were conducted by placing a linear polarizer in front of the entrance port of the camera.

Chemical Treatments. In the doping-dependent PL measurements, CTAC, deionized water, polystyrene (PS, molecular weight: 280,000), and nicotinamide adenine dinucleotide (NADH) were utilized as dopant molecules. The molecular adsorption was realized by immersing the WS_2 monolayer sample into the dopant solution for 1 h. The CTAC (1–5 mM) and NADH (2 mM) solutions were prepared in deionized water. The PS solution was prepared by dissolving PS (1 g)

into toluene (10 g) at 50 °C. The PS solution was spin-coated on the WS_2 monolayer sample and then removed by immersing the sample in a toluene bath for 4 h.

Finite-Difference Time-Domain (FDTD) Simulations. The electromagnetic simulations were performed using FDTD Solutions 8.19 (Ansys Lumerical). The Ag NR was modeled as a recumbent cylinder with two hemispherical ends. The WS_2 monolayer was modeled as a 1-nm-thick thin film. The dielectric function of Ag was calculated by fitting the experimental data of Palik. The refractive index of the glass substrate, WS_2, and air were set at 1.45, 4.71, and 1.00, respectively. A mesh size of 0.5 nm was employed in the simulations. A total-field scattered-field source at a fixed incidence angle of 64° relative to the surface normal was employed to simulate the dark-field scattering process from the individual nanostructures illuminated by a plane wave. An electric dipole was placed inside the WS_2 layer to simulate the exciton emissions. A power transmission box was used for calculating the scattering spectra and the intrinsic loss. The dipoles were placed at the end of the nanorod in the calculation of the intrinsic loss. The sign of power indicates the direction of energy flux. The power from the transmission box covering only a dipole without the Ag NR is 1. A near-field monitor was used to calculate the electric field and charge distributions at the resonance wavelengths. A three-dimensional power monitor was used to calculate the far-field radiation through near- to far-field projection.

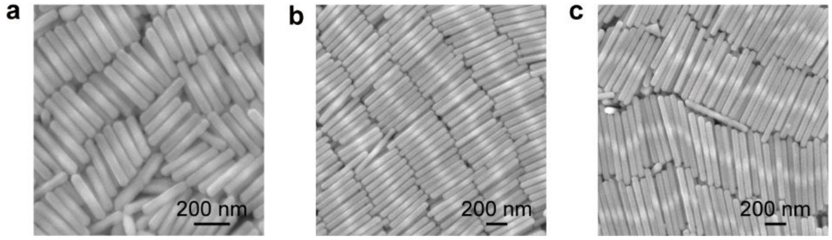

Figure S1. Ag NRs with different lengths. (a–c) SEM images of the Ag NR samples with average lengths of 310 ± 15 nm, 490 ± 21 nm, and 601 ± 38 nm, respectively. The three Ag NR samples have similar diameters, with an average of 58 ± 4 nm.

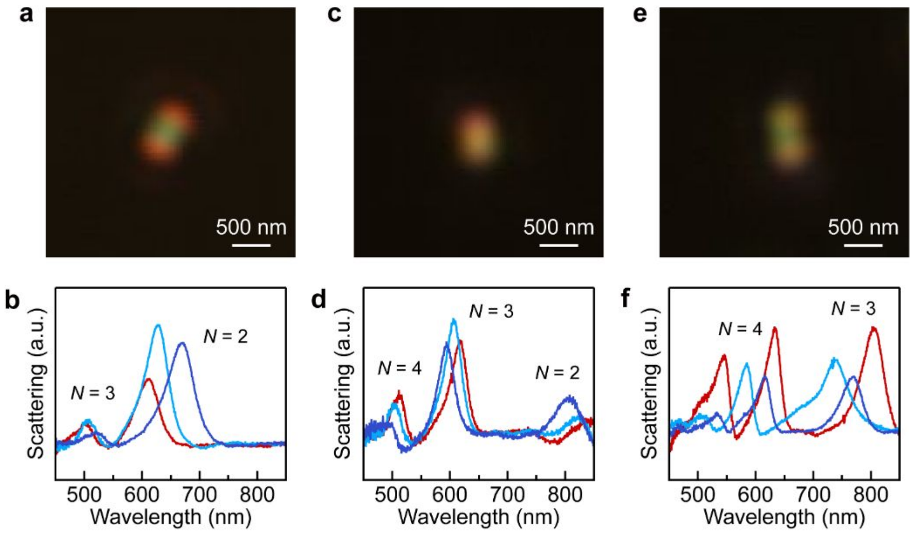

Figure S2. Ag NRs on a quartz substrate. (a and b) Dark-field scattering image (a) and spectra (b) of the Ag NR sample with an average length of 310 nm. The two plasmon peaks in the visible region are associated with the longitudinal quadrupole ($N = 2$) and octupole ($N = 3$) modes. The distinct far-field behaviors of the even and odd plasmon modes give rise to the red-green-red scattering pattern. (c–f) Dark-field scattering images and spectra obtained from two other Ag NR samples with average lengths of 490 nm (c and d) and 601 nm (e and f). The spectral positions of the multipole plasmon modes redshift as the length of the Ag NR is increased. When the length of the Ag NR is increased to ~490 nm, the $N = 4$ and $N = 3$ plasmon modes appear at ~500 nm and ~630 nm. The $N = 2$ plasmon mode redshifts to a longer wavelength close to the detection limit of our camera. The corresponding dark-field scattering image appears as a solid orange spot. When the length of the Ag NR is further increased to ~601 nm, the $N = 4$ plasmon mode redshifts to ~600 nm. The $N = 4$ plasmon mode can scatter towards the two ends of the Ag NR, leading to two bright spots in the scattering image.

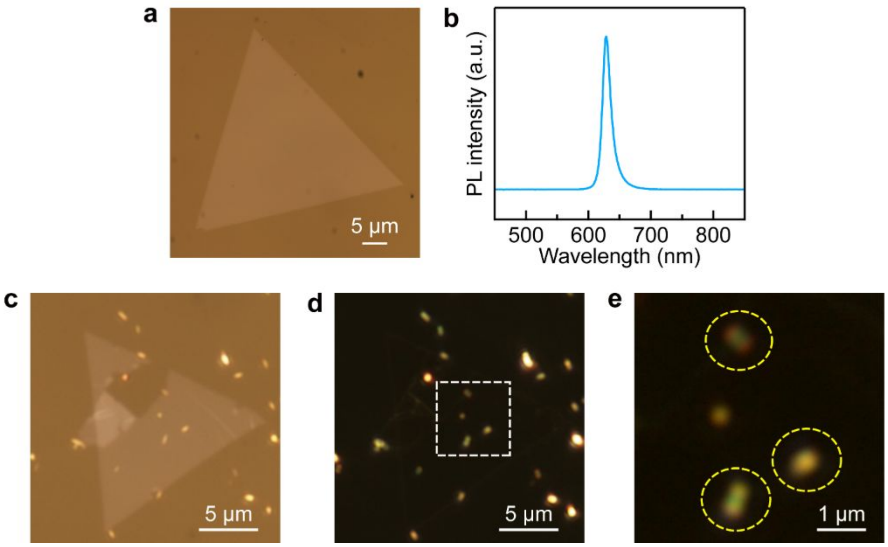

Figure S3. Ag NRs deposited on WS_2 monolayer. (a) Optical microscopy image of the WS_2 monolayer grown on a quartz substrate. (b) PL spectrum of the WS_2 monolayer. The exciton emissions of the grown WS_2 monolayer exhibit a pronounced peak at ~630 nm. The PL emissions contain contributions from both the A excitons and trions, exhibiting an asymmetric line shape. (c and d) Bright- (c) and dark-field (d) optical microscopy images after a mixture of the 310-nm-, 490-nm-, and 601-nm-long Ag NR samples was deposited on the WS_2 monolayer. (e) Magnified image of the area indicated by the white dashed box in (d). The scattering patterns indicated with the yellow dashed circles are similar to those of the Ag NRs on quartz substrates.

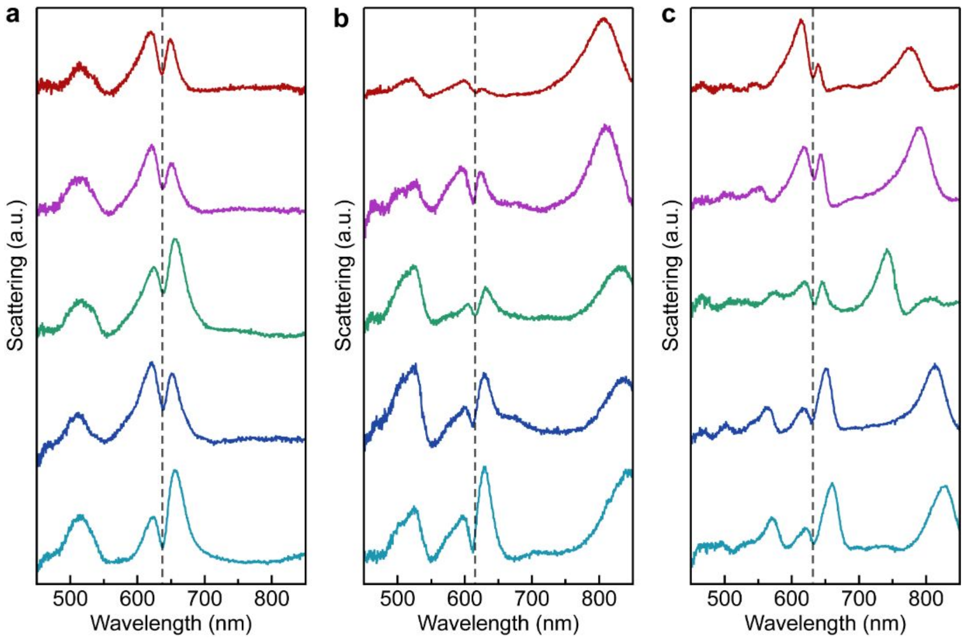

Figure S4. Resonance coupling in the (Ag NR)-on-WS_2 heterostructures. (a–c) Measured dark-field scattering spectra from the (Ag NR)-on-WS_2 heterostructures constructed from the three Ag NR samples with different lengths. The colorful solid lines represent the measured spectra. The vertical dashed lines indicate the scattering dips. The average lengths of the Ag NRs are ~310 nm (a), ~490 nm (b), and 601 nm (c). The excitons in the WS_2 monolayer are correspondingly coupled to the $N = 2$, $N = 3$, and $N = 4$ plasmon modes in the Ag NRs, respectively.

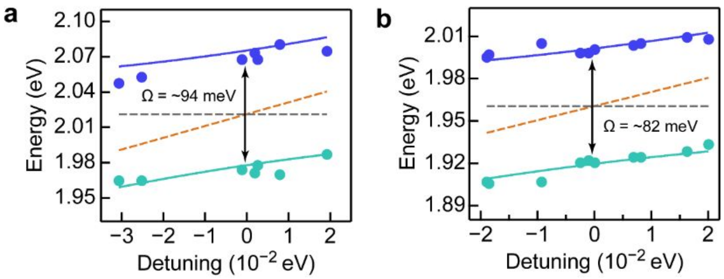

Figure S5. Energy dependence of the scattering peaks on the detuning. (a and b) High- (blue circles) and low-energy (green circles) hybrid modes (HEM and LEM) extracted from the scattering spectra of the Ag NRs on WS_2 monolayer with the NR lengths of ~490 nm (a) and 601 nm (b). The two solid lines represent the fitting results based on the coupled harmonic oscillator model. The orange and grey dashed lines show the plasmon resonance energy and the exciton transition energy, respectively. The energy difference between the HEM and LEM at zero detuning in (a) is ~94 meV, which is slightly higher than the overall loss of the system, $(\hbar\gamma_{ex}+\hbar\gamma_{pl})/2$. Herein $\hbar\gamma_{ex}$ = 52 meV is the linewidth of the exciton emissions in the WS_2 monolayer and $\hbar\gamma_{pl}$ = 128 meV is the average linewidth of the N = 3 plasmon mode of the Ag NRs. The splitting energy in (b) is ~82 meV, which is also larger than the overall loss of the system. The average linewidth of the N = 4 plasmon mode is ~103 meV.

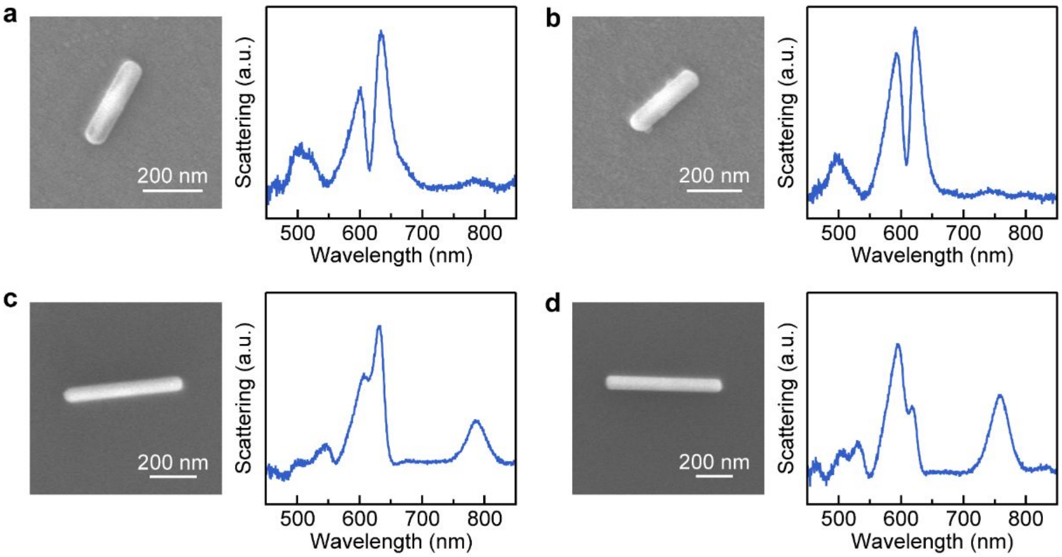

Figure S6. Ag NRs for the PL routing measurements. (a and b) SEM images and dark-field scattering spectra of two representative (Ag NR)-on-WS_2 heterostructures. The Ag NRs with a length of ~300 nm were deposited on the slightly doped WS_2 monolayer in (a) and highly doped WS_2 monolayer in (b) for the PL routing measurements. The $N = 2$ plasmon mode of the Ag NRs is seen to be resonantly coupled to the excitons. (c and d) Similar characterization for the 590-nm-lomg Ag NRs used in the optical measurements. The $N = 4$ plasmon mode of the Ag NRs is resonantly coupled to the excitons.

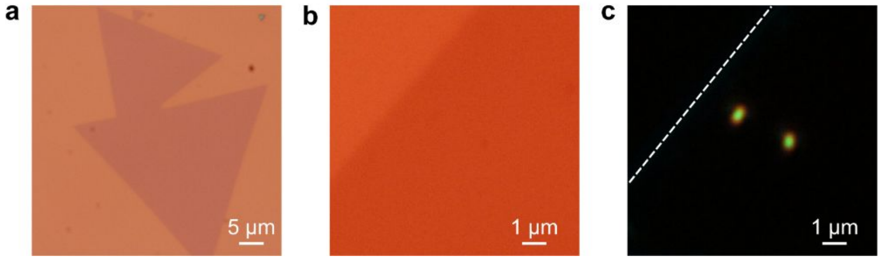

Figure S7. (Ag NR)-on-WS_2 heterostructures supported on Si/SiO_2 substrates. (a) Optical microscopy image of the WS_2 monolayer grown on a Si/SiO_2 (300 nm thickness) substrate. (b and c) Bright- and dark-field images of the (Ag NR)-on-WS_2 heterostructures. The white dashed line in (c) indicates the edge of the WS_2 monolayer.

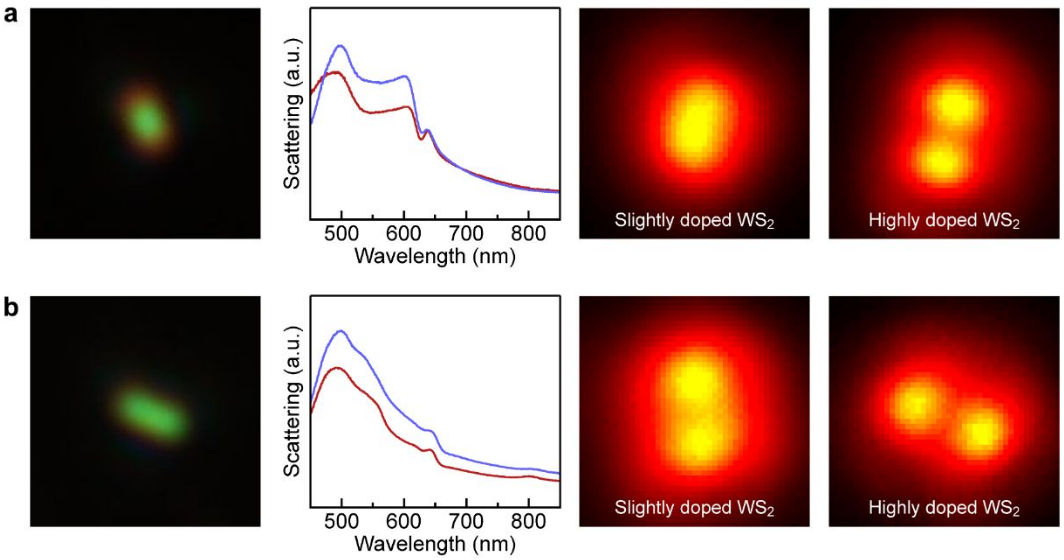

Figure S8. PL routing in the (Ag NR)-on-WS_2 heterostructures supported on Si/SiO_2 substrates. (a and b) Dark-field scattering and PL measurements of the individual (Ag NR)-on-WS_2 heterostructures constructed from the 310-nm- (a) and 601-nm-long (b) Ag NRs, respectively. The scattering peaks of the Ag NRs are largely enhanced in the short-wavelength region (450–550 nm) and suppressed at ~600 nm due to the interference from the thermal oxide layer. Although the thermal oxide layer altered the dark-field scattering images and spectra, the ∞-shaped PL emission patterns can still be clearly observed when the $N = 2$ and $N = 4$ plasmon modes are resonantly coupled to the excitons of the highly doped WS_2 monolayer.

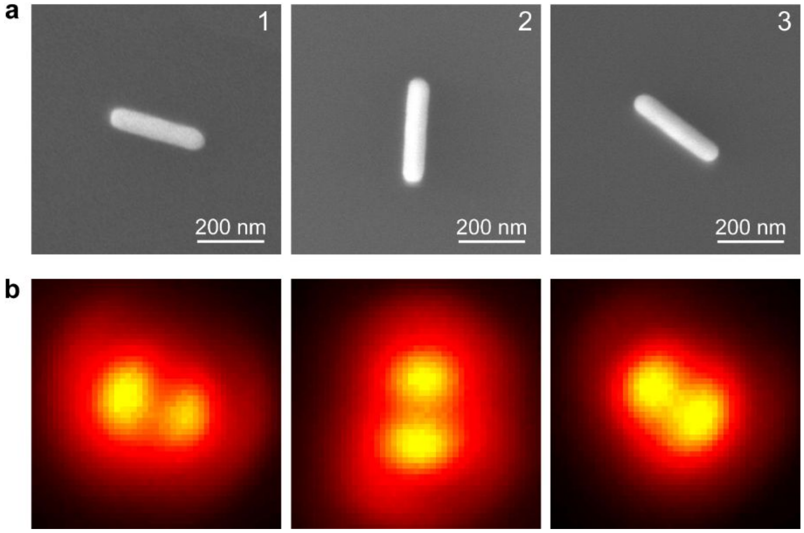

Figure S9. Correlation between the orientation of the Ag NR and the emission routing direction. (a) SEM images of three representative heterostructures constructed from the Ag NRs with different orientations. (b) Corresponding PL emission patterns showing two bright spots along the length axis of the Ag NR. The used Ag NR sample has an average length of 310 nm. The Ag NRs were deposited on the Si/SiO_2 substrates.

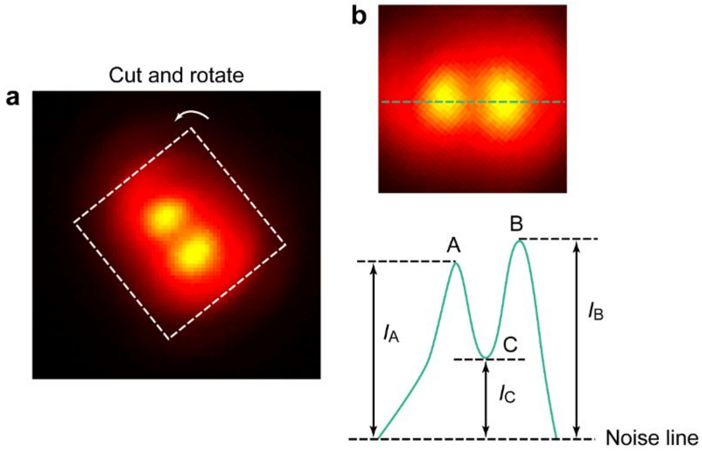

Figure S10. PL routing factor. (a) Schematic of the main procedure employed to obtain the longitudinal profile of the PL emission pattern. The region of interest, which is indicated with the white dashed box, at a certain angle was cut from the raw image and rotated. (b) Schematics showing the definition of the PL routing factor. The longitudinal intensity profile (bottom) was extracted from the emission pattern (top) as indicated by the green dashed line. I_A and I_B represent the intensity values at the two peak positions, while I_C denotes the intensity value at the valley position. The routing factor is then calculated as $[(I_A + I_B)/2]/I_C$.

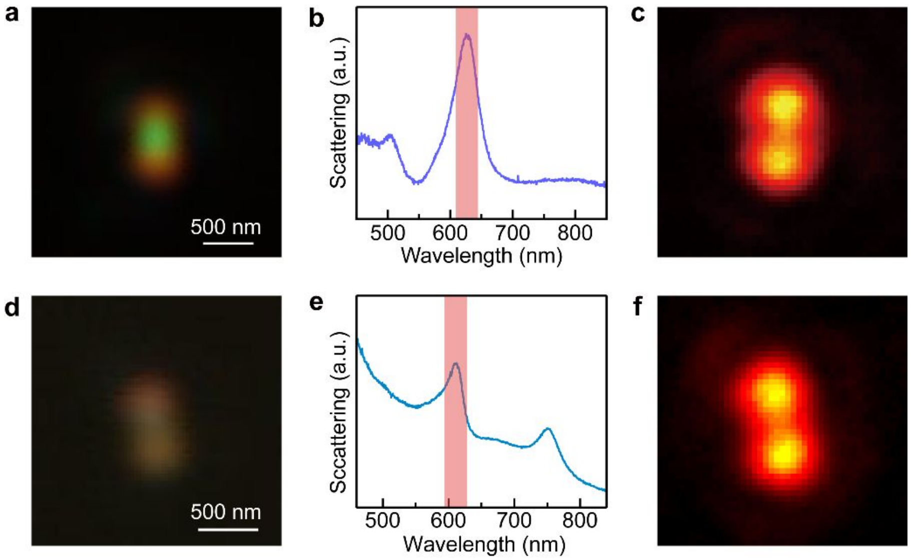

Figure S11. Scattering routing factor of the single Ag NRs. (a) Dark-field scattering image of an individual Ag NR captured by a color camera. (b) Dark-field scattering spectrum of the Ag NR. (c) Scattering image with use of an additional 630 nm band-pass filter in front of the charge-coupled device camera to pick out the $N = 2$ mode. The Ag NR is deposited on a quartz substrate. The scattering image is illustrated with a yellow hot color intensity scale. A scattering routing factor of 1.22 was estimated based on the scattering pattern. (d–f) Scattering image and spectrum of the Ag NR supporting the $N = 4$ mode at ~630 nm. The scattering routing factor for the $N = 4$ mode is 1.23.

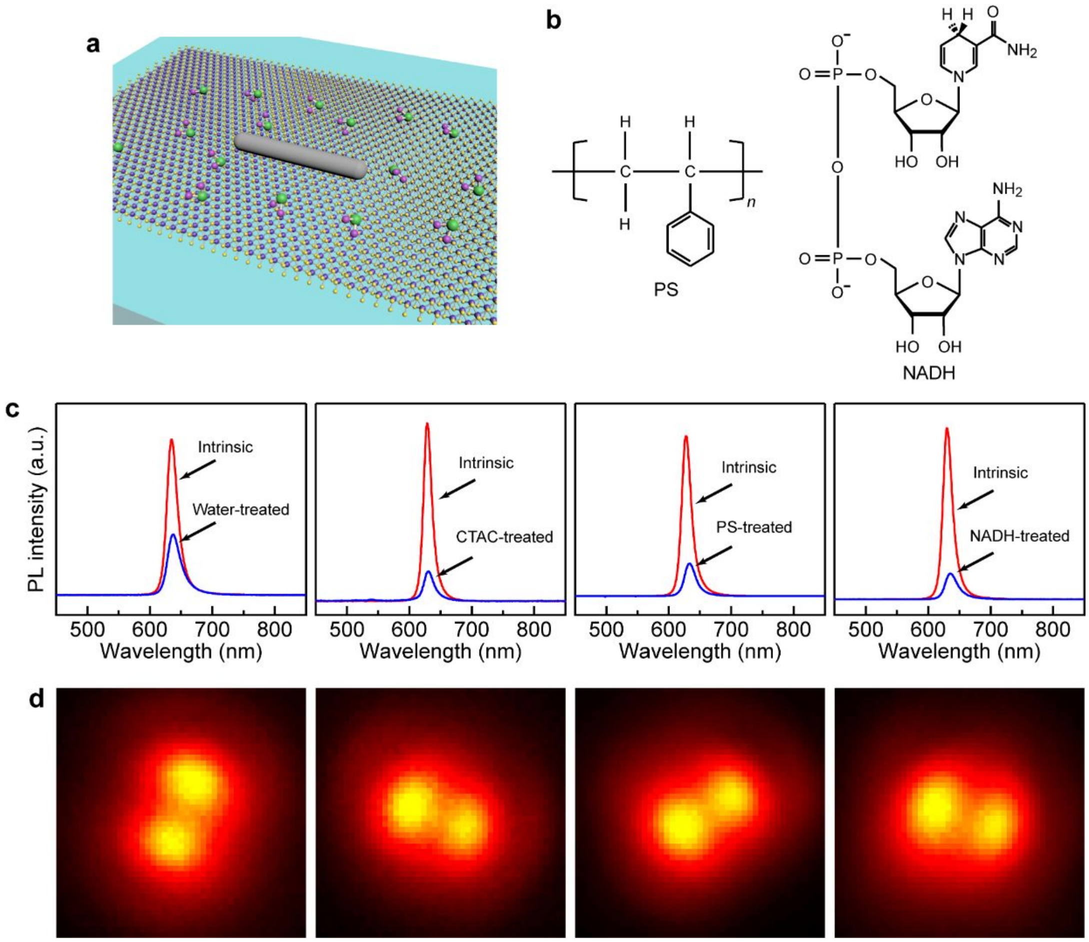

Figure S12. Chemical modification of the WS_2 monolayer for enhanced PL routing. (a) Schematic showing the chemical modification of the WS_2 monolayer. Four types of n-type dopants, including water, CTAC, PS, and NADH, were employed. Water molecules are drawn in the schematic as a representative of the n-type dopants. (b) Molecular structures of PS and NADH. (c) PL spectra measured before and after the chemical modification with the four types of dopants. (d) Representative PL patterns obtained after the doping processes. The results show that the routing of the exciton emissions can be realized in the doped WS_2 monolayer with various n-type dopants.

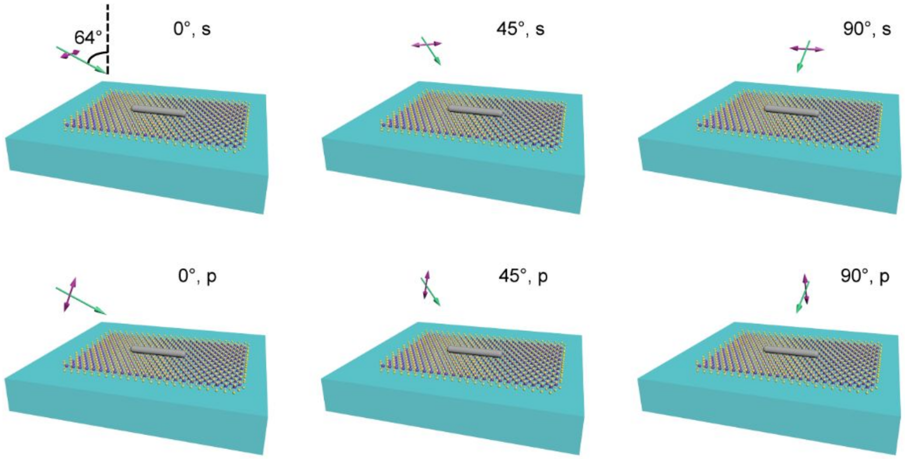

Figure S13. Excitation schemes in the FDTD simulations. The green arrow indicates the wavevector of the excitation light. The purple double-headed arrow shows the polarization direction of the excitation light. The incidence angle in our simulations was fixed at 64° relative to the surface normal according to the numerical aperture (0.9) of the dark-field objective used in our experiments.

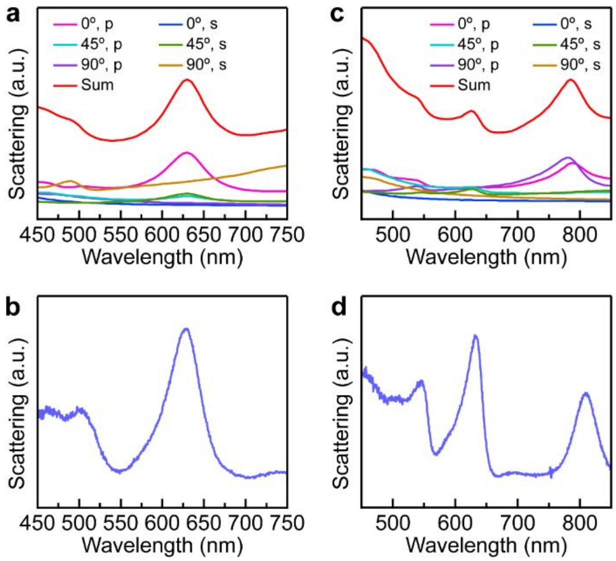

Figure S14. Simulated scattering spectra of the (Ag NR)-on-WS_2 heterostructures. (a–d) Simulated and measured scattering spectra of the (Ag NR)-on-WS_2 heterostructures constructed from the Ag NRs with two different lengths. The length/diameter of the Ag NRs were set at 305 nm/60 nm in (a) and 640 nm/60 nm in (c) in the FDTD simulations. The final simulated scattering spectra were obtained by summing up the spectra of the different excitation schemes. The measured scattering spectra of the Ag NRs with similar lengths were provided in (b and d) for comparison. It can be seen that the simulated scattering peaks fit well with the experimental ones.

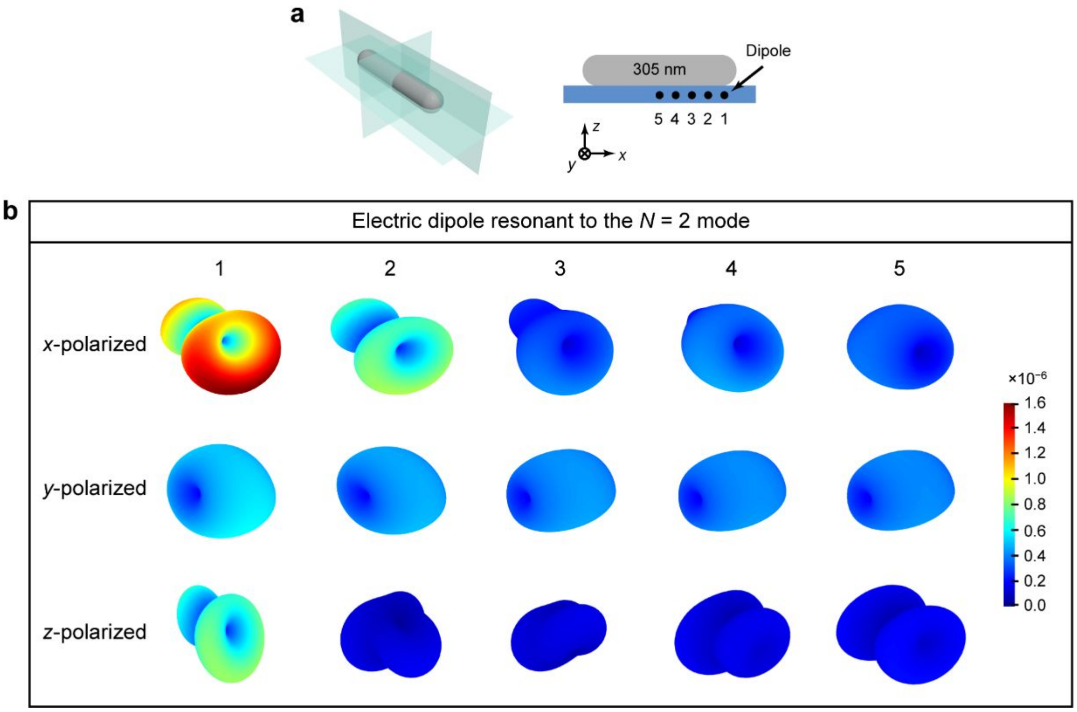

Figure S15. Far-field radiation when the $N = 2$ mode is resonant to the exciton emissions. (a) Schematics showing the spatial orientation of the Ag NR (left) and the emitter positions (right) relative to the Ag NR. The Ag NR has a length of 305 nm and a diameter of 60 nm. The dipoles, whose spectral position was set to 630 nm, were used to model the 2D excitons in the WS_2 monolayer. They were placed in the central plane of a 1-nm-thick film. The $N = 2$ plasmon mode is resonantly coupled to the exciton emissions in the WS_2 monolayer in this case. (b) Three-dimensional far-field radiation patterns. The far-field radiation patterns of the *x*- and *z*-polarized dipoles are efficiently modulated by the $N = 2$ mode. The emissions from the *x*-polarized dipole at Position 1 are the most significant due to the strong local field enhancement at the two ends of the nanorod and the fact that the dipole moment is aligned well with the local electric field of the longitudinal plasmon mode. The *y*-polarized dipoles cannot couple to the $N = 2$ plasmon mode. They might contribute to a uniform background.

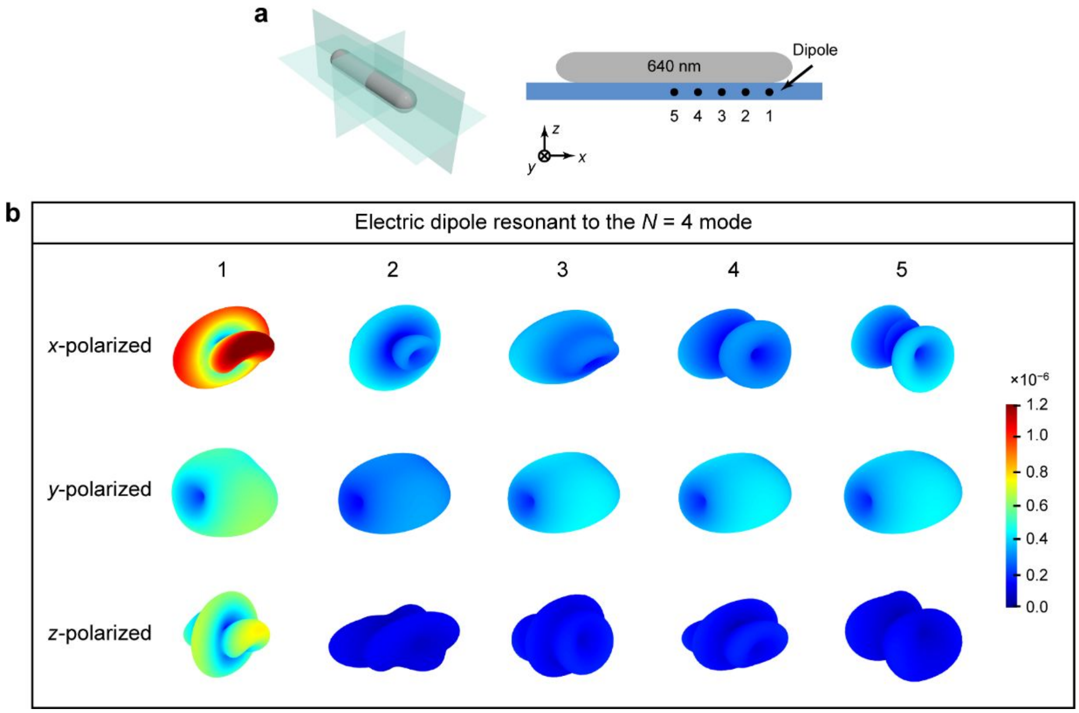

Figure S16. Far-field radiation when the $N = 4$ mode is resonant to the exciton emissions. (a) Schematics showing the spatial orientation of the Ag NR (left) and the emitter positions (right) relative to the Ag NR. The Ag NR has a length of 640 nm and a diameter of 60 nm. The emission wavelength of the dipoles was set to 630 nm. The $N = 4$ plasmon mode is resonantly coupled to the dipole emissions. (b) Three-dimensional far-field radiation patterns. The far-field radiation patterns of the *x*- and *z*-polarized dipoles are efficiently modulated by the $N = 4$ mode. The emissions from the *x*-polarized dipole at Position 1 are the most significant due to the strong local field enhancement at the two ends of the nanorod and the fact that the dipole moment is aligned well with the local electric field of the longitudinal plasmon mode. The *y*-polarized dipoles cannot couple to the $N = 4$ plasmon mode. They might contribute to a uniform background.

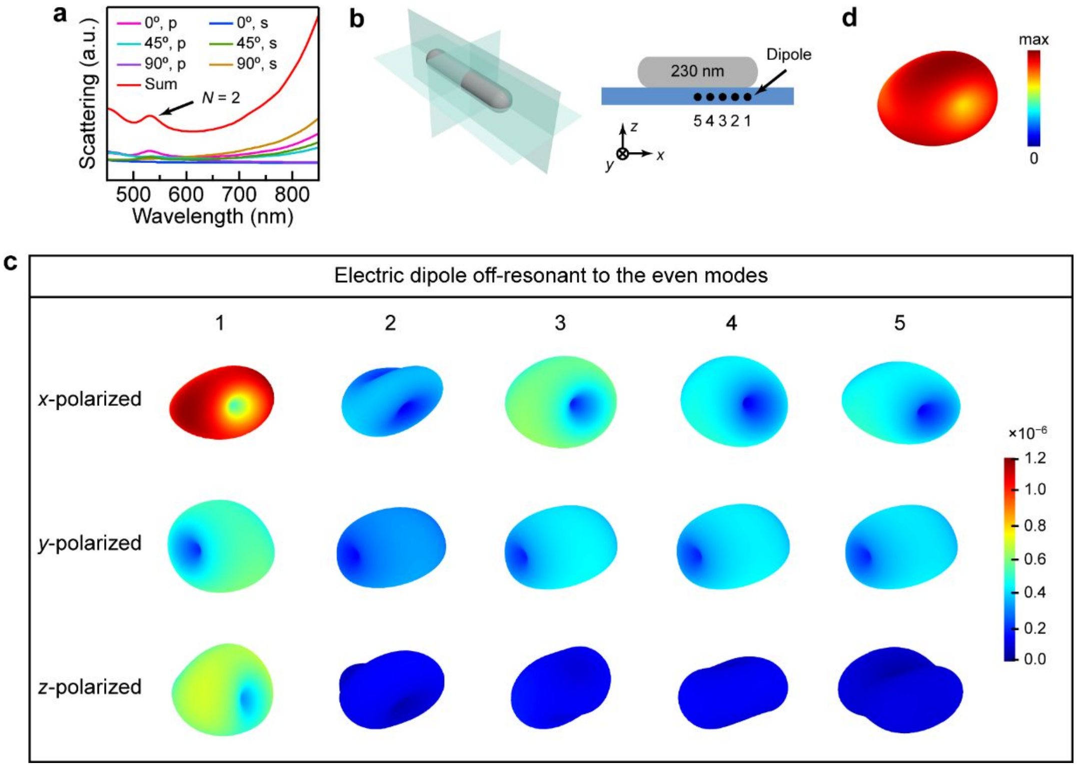

Figure S17. Far-field radiation when the dipole emissions are off-resonant to the even plasmon mode. (a) Simulated scattering spectra of the Ag NR with a length of 230 nm and a diameter of 60 nm. The $N = 2$ plasmon mode is located at 540 nm. (b) Schematics showing the spatial orientation of the Ag NR (left) and the emitter positions (right) relative to the Ag NR. The dipoles with an emission wavelength of 630 nm were placed in the central plane of a 1-nm-thick thin film used to model the WS_2 monolayer. The dipole emissions are off-resonant to the even plasmon mode in this case. (c) Three-dimensional far-field radiation patterns. The dipole emissions cannot be modulated by the plasmon mode in all cases. (d) Total far-field radiation arising from the dipole emissions from the five representative positions. For off-resonance lengths, the exciton emissions are hardly affected by the plasmon resonance and the pattern is rotationally symmetric.

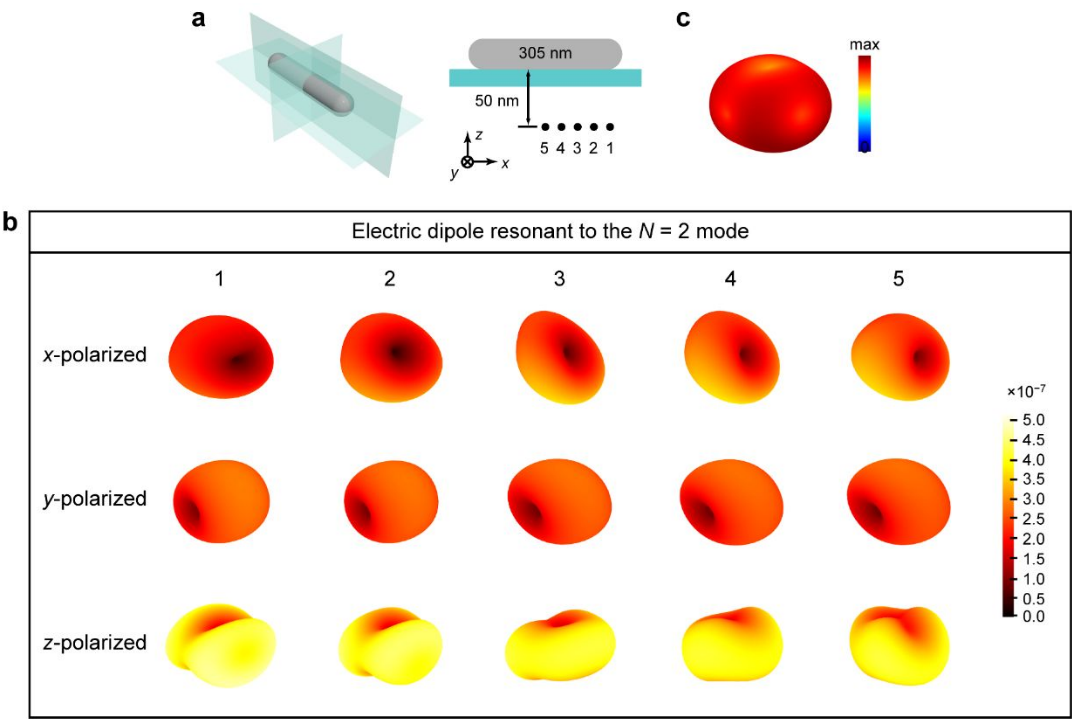

Figure S18. Far-field radiation when the dipoles are placed away from the Ag NR. (a) Schematics showing the spatial orientation of the Ag NR (left) and the emitter positions (right) relative to the Ag NR. The Ag NR has a length of 305 nm and a diameter of 60 nm. The dipoles with an emission wavelength of 630 nm were placed 50 nm away from the Ag NR. The thin layer (green) under the Ag NR was used to create a dielectric environment similar to the case with the WS_2 monolayer. (b) Three-dimensional far-field radiation patterns. The dipole emissions cannot be modulated by the plasmon mode in all cases. (c) Total far-field radiation arising from the dipole emissions from the five representative positions.

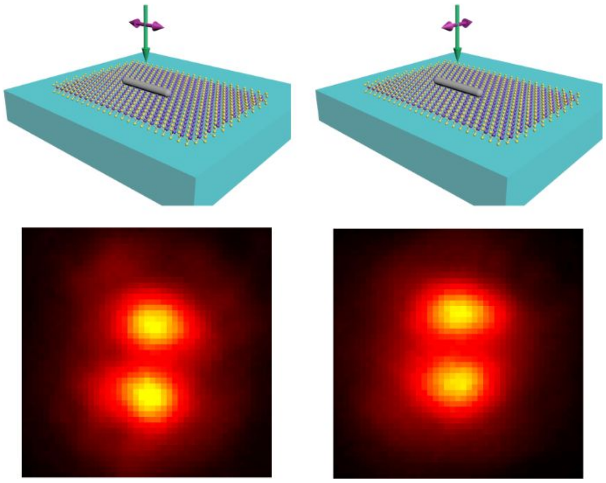

Figure S19. Excitation polarization dependence. The PL images were captured when the laser polarization was set to be parallel and perpendicular to the length axis of the Ag NR, respectively. The emission patterns obtained from the two cases are similar.

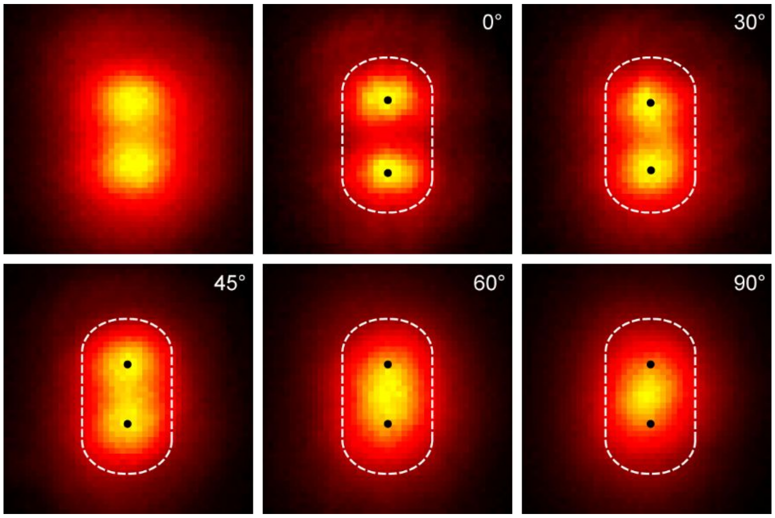

Figure S20. Polarization dependence of the PL routing effect. An analyzer was placed in front of the camera to investigate the polarization dependence of the PL emissions. The angle was set to 0° when the analyzer polarization is parallel to the length axis of the Ag NR. The PL images were captured at different analyzer angles. The laser polarization was always along the length axis of the Ag NR. The intensity values at the points indicated by the black dots were extracted and used to calculate the routing factors at different angles.

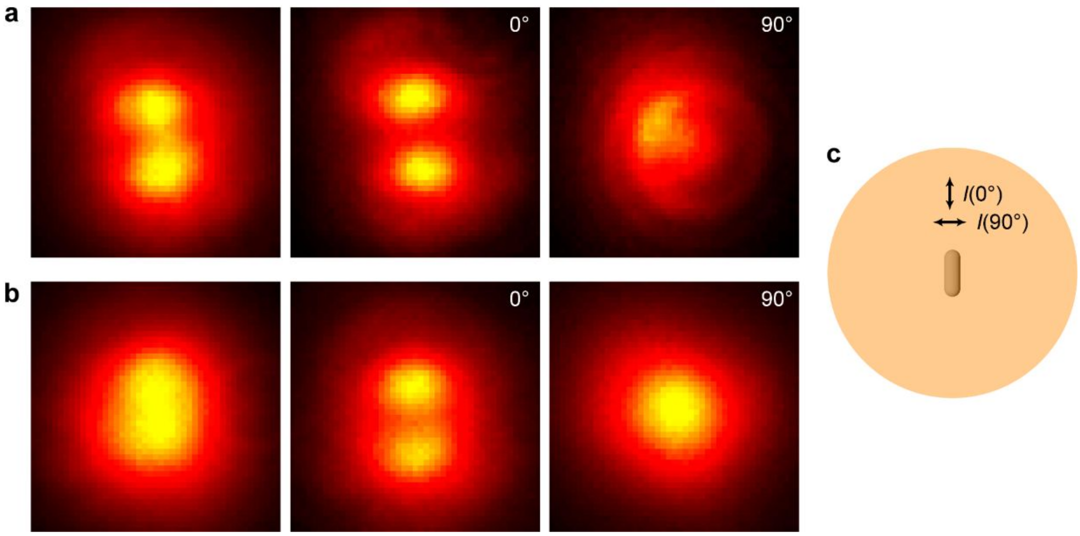

Figure S21. Effect of the PL enhancement. (a) A representative (Ag NR)-on-WS_2 heterostructure with a clear PL routing effect (left). The emission patterns were further captured with an analyzer in front of the camera. The analyzer polarization is 0° (middle) and 90° (right) relative to the length axis of the Ag NR. The PL images were illustrated on the same intensity scale. (b) A typical (Ag NR)-on-WS_2 heterostructure with a weak PL routing effect. The distinct routing effect was found to appear when the emission intensity at the polarization angle 0° is much higher than that at 90°. The 0° polarization picks out the fluorescence signal modulated by the longitudinal plasmon mode of the Ag NRs. The above results strongly indicate that the PL routing effect can only be observed in the far-field when the PL enhancement of the exciton emissions are large enough. (c) Schematic showing the calculation of the PL enhancement factor. The black arrows show the polarization directions of the analyzer in front of the entrance of the camera. I(0°) and I(90°) represent the integrated PL intensities obtained at the angles of 0° and 90°, representing the exciton emissions modulated by the plasmon resonance and the background emission from the bare WS_2 monolayer. The PL enhancement factor is defined as $[(I(0°)-I(90°))/S]/[I(90°)/S_0]$, where S_0 is the area of the laser spot with a diameter of ~1 μm, S is the area of the WS_2 monolayer under an individual nanorod in the (Ag NR)-on-WS_2 heterostructure, which is approximated to be $S = d \times l$, with d and l representing the diameter and the length of the nanorod, respectively.

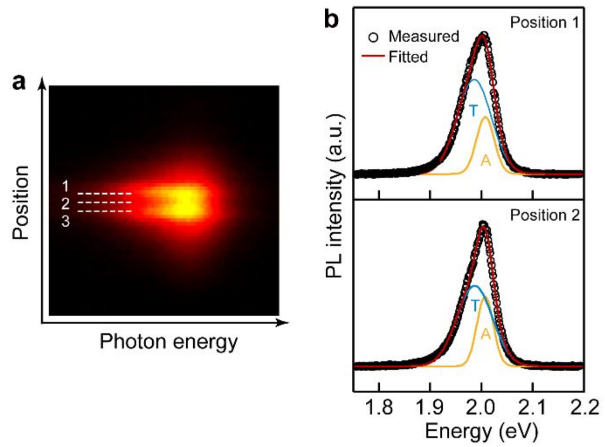

Figure S22. Contribution of the A excitons and trions in the emission routing. (a) PL image obtained in the grating mode. The *x* axis of the PL image represents the emission photon energy, while the *y* axis shows the spatial distribution of the PL emissions. The Ag NR was carefully oriented parallel to the slit in front of the spectrometer. It is aligned vertically in the image. (b) PL spectra extracted from different positions along the *y* axis in (a). The PL spectra were fitted using Gaussian peaks. (c) Integrated PL intensities of the A excitons and trions. The fitting results clearly show that the A excitons and trions exhibit distinct spatial distributions. The trion emissions are routed towards the two ends of the Ag NR, while the routing effect of the A excitons is weak.

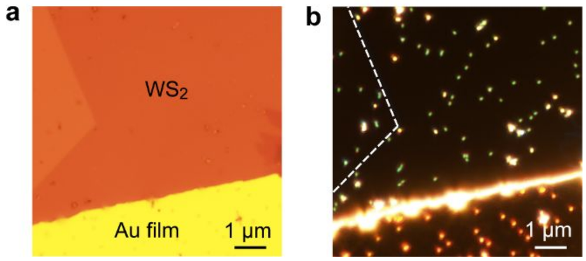

Figure S23. Electrically biased (Ag NR)-on-WS_2 heterostructure. (a) Bright-field optical microscopy image of the electrically biased (Ag NR)-on-WS_2 heterostructures supported on a Si/SiO_2 substrate. The Ag NR sample with an average length of 310 nm was employed. The WS_2

monolayer was electrically contacted to a piece of Au film and grounded. The Si layer was used as the back gate. (b) Dark-field scattering image of the same area in (a). The white dashed line shows the edge of the WS_2 monolayer.

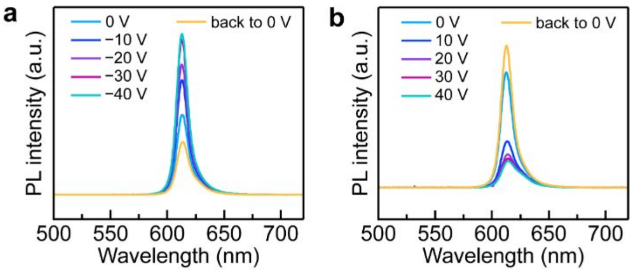

Figure S24. Electrically modulated PL emissions of the WS_2 monolayer. (a) Evolution of the PL emissions on negative gate voltages. The holes are injected to the WS_2 monolayer when the gate is negatively biased. The naturally n-type doped WS_2 monolayer is compensated, leading to an enhanced quantum yield. The PL intensity of the WS_2 monolayer is therefore increased. The PL emission was recovered when the gate voltage was reset to 0 V. (b) Evolution of the PL emission on positive gate voltages. The PL emissions of the WS_2 monolayer is suppressed due to n-type doping.

Table S1. Simulated Powers Leaving the Transmission Boxes Covering the Different Parts of the (Ag NR)-on-WS_2 Heterostructure

power leaving the transmission box	dipole	Ag NR	dipole + Ag NR
x-polarized	2189	-2117	4
y-polarized	1163	-1162	0.3
z-polarized	1431	-1321	2.4

REFERENCES

(1) Zhuo, X. L.; Zhu, X. Z.; Li, Q.; Yang, Z.; Wang, J. F. Gold nanobipyramid-directed growth of length-variable silver nanorods with multipolar plasmon resonances. *ACS Nano* **2015**, *9*, 7523–7535.

(2) Li, Q.; Zhuo, X. L.; Li, S.; Ruan, Q. F.; Xu, Q.-H.; Wang, J. F. Production of monodisperse gold nanobipyramids with number percentages approaching 100% and evaluation of their plasmonic properties. *Adv. Opt. Mater.* **2015**, *3*, 801–812.

(3) Zhang, Z. W.; Chen, P.; Duan, X. D.; Zang, K. T.; Luo, J.; Duan, X. F. Robust epitaxial growth of two-dimensional heterostructures, multiheterostructures, and superlattices. *Science* **2017**, *357*, 788–792.
